# Do attributes of persons with chronic kidney disease differ in low-income and middle-income countries compared with high-income countries? Evidence from population-based data in six countries

**DOI:** 10.1136/bmjgh-2017-000453

**Published:** 2017-10-09

**Authors:** Shuchi Anand, Yuanchao Zheng, Maria E Montez-Rath, Wang Jin Wei, Norberto Perico, Sergio Carminati, KM Venkat Narayan, Nikhil Tandon, Viswanathan Mohan, Vivekanand Jha, Luxia Zhang, Giuseppe Remuzzi, Dorairaj Prabahkaran, Glenn M Chertow

**Affiliations:** 1 Division of Nephrology, Stanford University School of Medicine, Stanford, California, USA; 2 Renal Division, Department of Medicine, Peking University First Hospital, Beijing, China; 3 Peking University Institute of Nephrology, Beijing, China; 4 Department of Biomedical and Clinical Sciences, Istitutodi Ricerche Farmacologiche Mario Negri, University of Milan, Milan, Province of Milan, Italy; 5 Global Health and Epidemiology, Emory University Rollins School of Public Health, Atlanta, Georgia, USA; 6 All India Institute of Medical Sciences, New Delhi, India; 7 Dr. Mohan’s Diabetes Specialities Centre, Endocrinology, Madras Diabetes Research Foundation, Chennai, Tamil Naidu, India; 8 George Institute for Global Health India, University of Oxford, New Delhi, India; 9 Public Heath Foundation of India, New Delhi, India

**Keywords:** epidemiology, indices of health and disease and standardisation of rates, cross-sectional survey

## Abstract

Kidney biopsies to elucidate the cause of chronic kidney disease (CKD) are performed in a minority of persons with CKD living in high-income countries, since associated conditions—that is, diabetes mellitus, vascular disease or obesity with pre-diabetes, prehypertension or dyslipidaemia—can inform management targeted at slowing CKD progression in a majority. However, attributes of CKD may differ substantially among persons living in low-income and middle-income countries (LMICs). We used data from population or community-based studies from five LMICs (China, urban India, Moldova, Nepal and Nigeria) to determine what proportion of persons with CKD living in diverse regions fit one of the three major clinical profiles, with data from the US National Health Nutrition and Examination Survey as reference. In the USA, urban India and Moldova, 79.0%–83.9%; in China and Nepal, 62.4%–66.7% and in Nigeria, 51.6% persons with CKD fit one of three established risk profiles. Diabetes was most common in urban India and vascular disease in Moldova (50.7% and 33.2% of persons with CKD in urban India and Moldova, respectively). In Nigeria, 17.8% of persons with CKD without established risk factors had albuminuria ≥300 mg/g, the highest proportion in any country. While the majority of persons with CKD in LMICs fit into one of three established risk profiles, the proportion of persons who have CKD without established risk factors is higher than in the USA. These findings can inform tailored CKD detection and management systems and highlight the importance of studying potential causes and outcomes of CKD without established risk factors in LMICs.

**Key questions**

**What is already known about this topic?**

- A growing number of studies have evaluated the prevalence of chronic kidney disease (CKD) in low-income and middle-income countries; thus, we can estimate how many persons have CKD in many low-income and middle-income countries.
- We can say less about who has CKD: no comparisons have been performed to determine whether attributes of persons with CKD living in low-income and middle-income countries differ from those persons with CKD living in high-income countries.
- Nephrologists perform kidney biopsies to elucidate cause in a minority of persons with CKD. Associated conditions—primarily, diabetes mellitus, vascular disease or obesity with pre-diabetes, prehypertension or dyslipidaemia—can offer a sufficient explanation to inform management targeted at slowing CKD progression.

**What are the new findings?**

- We find that a majority of persons with CKD, about two-thirds, living in five low-income and middle-income countries fit one of the three predefined risk factor profiles.
- The relative proportion of each differs substantially, with diabetes most common in urban India, vascular disease most common in Moldova and obesity with prehypertension, pre-diabetes or dyslipidaemia most common in China.
- In contrast to the USA, where nearly 85% of persons with CKD have one of the three associated conditions, more people are in the ‘CKD without established risk factors’ category, especially in Nigeria, where the high proportion of persons with significant albuminuria suggested the important contribution of apolipoprotein-1 nephropathy.

**Recommendations for policy**

- This approach of describing attributes of persons with CKD can help to tailor screening, detection and management strategies within each country.
- Our approach also highlights the need to study correlates and outcomes of persons with CKD without established risk factors in low-income and middle-income countries; it is possible that these persons have novel risk factors for CKD, or alternatively, we need to apply different thresholds to define CKD in diverse populations.

## Introduction

A growing number of studies in countries around the world indicate that roughly 10% of adults have evidence of chronic kidney disease (CKD),^1 2^ a finding that holds true across regions and income categories. While we know more about *how many* persons have CKD, we know less about *who* develops CKD in low-income and middle-income countries (LMICs) and whether the attributes of persons who develop CKD in LMICs differ substantially from those in high-income countries (HICs). In the USA^3^ and other HICs,^4 5^ associated diabetes mellitus, vascular disease and/or obesity are present in a majority of persons with CKD.

Many experts speculate that attributes of persons with CKD *do* differ substantially in LMIC, which experience a higher burden of infectious disease, urinary stone disease, low birthweight and, in some regions, primary glomerular disease.^6 7^ In several regions, a form of tubulointerstitial nephritis deemed CKD of unknown aetiology has been increasingly recognised, although its cause or causes have remained elusive.^8^ Determining what proportion of persons with evidence of CKD living in LMIC fit profiles similar to those of persons with CKD in HIC could be the first step towards understanding whether CKD screening strategies in LMIC need to target different populations and whether preventive efforts should be directed to different types of risk factors than in HIC.

Unlike HICs where population-based studies are often performed on an ongoing basis, surveillance systems to evaluate trends in, and/or attributes of, chronic diseases are weak in LMICs. We found existing data on characteristics of persons with CKD from population-based studies in five low, lower-middle and upper-middle income countries: China, India, Moldova, Nepal and Nigeria. Across this range of settings and in comparison with data from the USA, we describe the proportion of persons with CKD fitting one of three profiles—diabetes mellitus, vascular disease and obesity with pre-diabetes, prehypertension or dyslipidaemia—and the characteristics of persons who do not share any of these attributes.

## Methods

We used data from six population-based studies, three of which (from Nepal, Nigeria and Moldova) were conducted with support from the International Society of Nephrology (ISN) and using a standard data collection template (ISN Kidney Disease Data Center (KDDC)).^9^ Data from urban India (Center for Cardiometabolic Risk Reduction in South Asia (CARRS) surveillance study)^10 11^ and China^12^ are drawn from surveys with representative sampling techniques; the publicly available US National Health and Nutrition Examination Survey (NHANES)^13^ 1999–2006 cohorts provides the comparator data set (table 1). In generating our analytic sample, we excluded individuals who were younger than 20 years and with missing data on urine *or* serum markers of CKD. All studies obtained institutional review board approval at their coordinating institutions and adhered to principles outlined in the Declaration of Helsinki.

**Table 1:** Design and sample size of studies included in analysis

Study	Design	Representative sampling	Years	Sample size used in analysis*	Sample size of study
US NHANES	Repeated cross-sectional, national survey	Y	2009–2014	7323	7323
China	National cohort	Y	2009–2010	47 191	47 204
India CARRS	Cohort study; population in Delhi and Chennai	Y	2011	10 205	12 271
ISN KDDC Moldova	General population invited to two primary healthcare units in Chisinau and Ialoveni	N	2006–2008	1384	2105
ISN KDDC Nepal	General population invited to temporary or permanent centres	N	2006–2011	19 959	21 809
ISN KDDC Nigeria	General population invited to temporary or permanent centres	N	2008–2009	1911	1939

*Sample size for all studies represents persons ≥20 years of age and with available data on kidney disease markers; additionally, in NHANES, it represents persons who participated in the fasting laboratory draw.

CARRS, Center for Cardiometabolic Risk Reduction in South Asia; ISN, International Society of Nephrology; KDDC, Kidney Disease Data Center; NHANES, National Health and Nutrition Examination Survey.

### Definition of CKD

With the 2012 Kidney Disease Improving Global Outcomes guidelines^14^ as a reference, we defined a participant as having CKD with albuminuria (albumin:creatinine ratio (ACR) ≥30 mg/g creatinine and/or Chronic Kidney Disease Epidemiology Collaboration (CKD-EPI)^15^ estimated glomerular filtration rate (eGFR) <60 mL/min/1.73 m^2^). We used the race-adjusted coefficients for eGFR calculation for the Nigeria data. When incorporating data from the ISN KDDC, we assessed albuminuria as an approximate equivalent of ACR using an algorithm that relies on ACR, protein:creatinine ratio and protein dipstick, strictly in this order.^9 16^ We categorised persons having albuminuria if ACR ≥30 mg/mmol creatinine, protein:creatinine ratio ≥150 mg/g or protein dipstick 1+ or above. Protein dipstick was most often used in Nepal (online supplementary table 1).

10.1136/bmjgh-2017-000453.supp1 Supplementary file 1

### Definition of profiles

On the basis of published data from the US Renal Data System^3^ on clinical attributes of persons with CKD in the USA, we created four profiles:

1. Diabetes: fasting glucose ≥7.0 mmol/L or glycosylated haemoglobin (A1c≥6.5%) or self-reported diabetes.^17^
2. Vascular disease: self-reported history of coronary artery disease, myocardial infarction, congestive heart failure, stroke or current or former regular smoking.
3. Obesity with an additional cardiovascular risk factor: we categorised a person as obese if he or she had abnormal waist circumference as defined by sex-specific and ethnicity-specific cut-offs.^18^ In addition, to fit in this profile, he or she had either fasting glucose (5.6–6.9 mmol/L), haemoglobin A1c (5.7%–6.4%), measured systolic blood pressure ≥130 mm Hg and diastolic ≥85 mm Hg, triglycerides ≥1.7 mmol/L or low high-density lipoprotein <1.0 mg/dL for men or <1.3 mg/dL for women.^18^
4. CKD without established risk factors: persons without any risk factors as outlined in profiles 1–3.

Online supplementary table 2 lists available data and associated measurement methods for profiles across studies; online supplementary table 3 lists acceptable ranges for laboratory tests used when defining profiles.

### Statistical analyses

For all harmonised data elements among the six cohorts (online supplementary table 3), we report means (±SD) and proportions as appropriate. For the three representative studies (from China, urban India and the USA), we report N, means and proportions. After categorising persons into mutually exclusive profiles in sequence from profiles 1 to 3, we report the relative proportion of persons with CKD in one of the four profiles. Since haemoglobin A1c was available in only two studies, we performed sensitivity analyses using fasting glucose thresholds alone in these studies.

In considering missing data, all non-affirmative responses were treated as absent condition for comorbidities dependent on self-report.^19^ For measured markers (eg, fasting glucose or waist circumference), the missingness was low among persons with CKD (<10%, see online supplementary table 4) and was considered in the following way: in calculating the proportion with diabetes (profile 1), for example, if a participant was missing haemoglobin A1c or missing fasting glucose and did not self-report diabetes, he/she was labelled as having a ‘missing’ diabetes status. However, if a participant had an abnormal value for either fasting glucose or haemoglobin A1c, he/she was treated as having diabetes. We evaluated and report any differences in missingness of data elements required to categorise a participant with CKD in profiles 1–3 across the studies. All statistics for NHANES, CARRS and China studies take into account complex survey design including subpopulation methods when appropriate. We used SAS V.9.4 (Cary, North Carolina).

## Results

Table 1 briefly describes methods of each study included in this analysis. Table 2A describes participants with available information on CKD markers (urine albumin and serum creatinine data) in the studies with representative sampling design. Mean age was higher in the USA, as were self-reported rates of smoking and cardiovascular disease. More people in China and urban India had blood pressure measured in the highest categories (≥140 mm Hg systolic or ≥90 mm Hg), although prevalence of hypertension was similar to the USA. Diabetes prevalence and proportion of people in the highest fasting glucose categories was highest in urban India. Unadjusted CKD prevalence ranged from 8.09% to 15.15%.

**Table 2A:** Characteristics of persons living in USA, China and urban India, as captured by studies performed using a representative sample

	USA	China	Urban India
Age (years)	47.3 (0.4)	42.3 (0.2)	41.9 (0.7)
20–40 (%)	38.5	48.7	50.1
41–60 (%)	37.4	35.7	42.8
61+ (%)	24.1	14.2	7.1
Missing (%)		1.3	
Female (%)	51.9	49.7	52.1
Current or former smoker (%)	39.4	22.8	14.7
History of cardiovascular disease (%)*	8.2	1.7	3.0
Waist circumference (cm)	99.1 (0.3)	80.6 (0.1)	86.7 (0.4)
Missing (%)	2.8	0.9	3.3
Abnormal (%)†	54.7	51.2	55.7
Systolic blood pressure (mm Hg)	120.6 (0.3)	125.7 (0.2)	124.3 (0.7)
Missing (%)	3.2	0.9	1.8
<130 (%)	73.5	62.9	66.9
130≤140 (%)	11.9	14.4	14.4
≥140 (%)	11.5	21.9	17.0
Diastolic blood pressure (mm Hg)	69.7 (0.3)	80.50 (0.1)	82.77 (0.3)
Missing (%)	3.6	0.9	1.8
<85 (%)	89.1	68.7	59.7
85≤90 (%)	3.9	9.6	14.7
≥90 (%)	3.4	20.9	23.9
Fasting glucose (mg/dL)	104.8 (0.5)	94.23 (0.2)	110.96 (0.9)
Missing (%)	0.0	0.6	0.2
<5.6 (%)	53.9	76.0	50.1
5.6≤6.9 (%)	36.8	19.5	35.9
≥7.0 (%)	9.3	4.0	13.7
Haemoglobin A1c (%)	5.6 (0.2)		6.30 (0.1)
Missing (%)	0.2	−	0.9
<5.7 (%)	67.4	−	34.3
5.7–6.4 (%)	24.0	−	39.2
≥6.5 (%)	8.4	−	25.6
Diabetes (%)‡	13.2	5.0	28.2
Hypertension (%)‡	37.7	30.3	34.6
CKD (%)	15.2	13.7	8.1

Values are reported as mean (SE) or per cent as appropriate, using complex survey methods.

*Includes stroke, peripheral arterial disease, congestive heart failure, myocardial infarction/coronary artery disease.

†Abnormal waist circumference as based on ethnicity-specific cut-offs.^18^

‡A participant is defined as having diabetes if he or she is in the upper fasting glucose or A1c categories or self-reported physician diagnosis of diabetes. A participant is defined as having hypertension if he or she is in the upper systolic or diastolic blood pressure categories or self-reported physician diagnosis of hypertension.

CKD, chronic kidney disease.

**Table 2B:** Characteristics of participants in population-based International Society of Nephrology Kidney Disease Data Center studies in Moldova, Nepal and Nigeria

	Moldova	Nepal	Nigeria
Age (years)	51.2 (14.0)	42.0 (15.2)	44.4 (13.2)
20–40 (%)	24.9	52.5	44.2
41–60 (%)	46.0	34.9	42.8
61+ (%)	29.1	12.6	13.0
Female (%)	70.7	62.0	63.4
Current or former smoker (%)	19.0	23.2	6.9
History of cardiovascular disease (%)*	25.6	1.5	0.4
Waist circumference (cm)	93.0 (13.4)	79.5 (11.3)	83.62 (11.86)
Missing (%)	7.2	0.2	0.2
Abnormal (%)†	70.2	36.9	44.0
Systolic blood pressure (mm Hg)	132.3 (20.9)	122.2 (18.8)	124.39 (21.15)
Missing (%)	2.2	0.0	0.5
<130 (%)	39.4	64.8	60.4
130≤140 (%)	18.4	15.4	14.5
≥140 (%)	40.0	19.8	24.5
Diastolic blood pressure (mm Hg)	84.2 (11.2)	80.9 (12.2)	81.3 (13.7)
Missing (%)	2.2	0.0	0.5
<85 (%)	50.1	66.2	66.9
85≤90 (%)	4.3	1.6	0.5
≥90 (%)	43.4	32.2	32.0
Fasting glucose (mmol/L)	86.3 (41.5)	88.3 (32.8)	85.6 (27.9)
Missing (%)	3.9	0.2	15.2
<5.6 (%)	80.6	81.4	75.3
5.6≤6.9 (%)	7.2	12.2	6.4
≥7.0 (%)	8.2	6.2	3.1
Diabetes (%)‡	12.8	8.9	6.0
Hypertension (%)‡	57.9	38.3	39.5
CKD (%)	25.4	20.9	23.1

Values are reported as mean (SE) or per cent as appropriate.

*Includes stroke, peripheral arterial disease, congestive heart failure, myocardial infarction/coronary artery disease.

†Abnormal waist circumference as based on sex-specific and ethnicity-specific cut-offs.^18^

‡A participant is defined as having diabetes if he or she is in the upper fasting glucose or A1c categories or self-reported physician diagnosis of diabetes. A participant is defined as having hypertension if he or she is in the upper systolic or diastolic blood pressure categories or self-reported physician diagnosis of hypertension.^9^

CKD, chronic kidney disease.

Table 2B describes participants in the community-based ISN KDDC screening studies. Women were more likely to participate; in Moldova, participants were older and more likely to report cardiovascular disease and have hypertension or diabetes. Participants in Nepal reported the highest rates of smoking. At 20.9%–25.4%, unadjusted CKD prevalence was higher in the ISN studies than in the population-representative design studies.

Online supplementary table 4 reports these characteristics for persons categorised as having CKD. In the USA, urban India, China and Moldova, albuminuria alone was the most common form of CKD, whereas in Nepal and Nigeria, eGFR <60 mL/min/1.73 m^2^ (with and without albuminuria) was most common. Significant albuminuria (≥ 300 mg/g) was most common in Nigeria.

### Profiles of CKD

**Figure 1:**
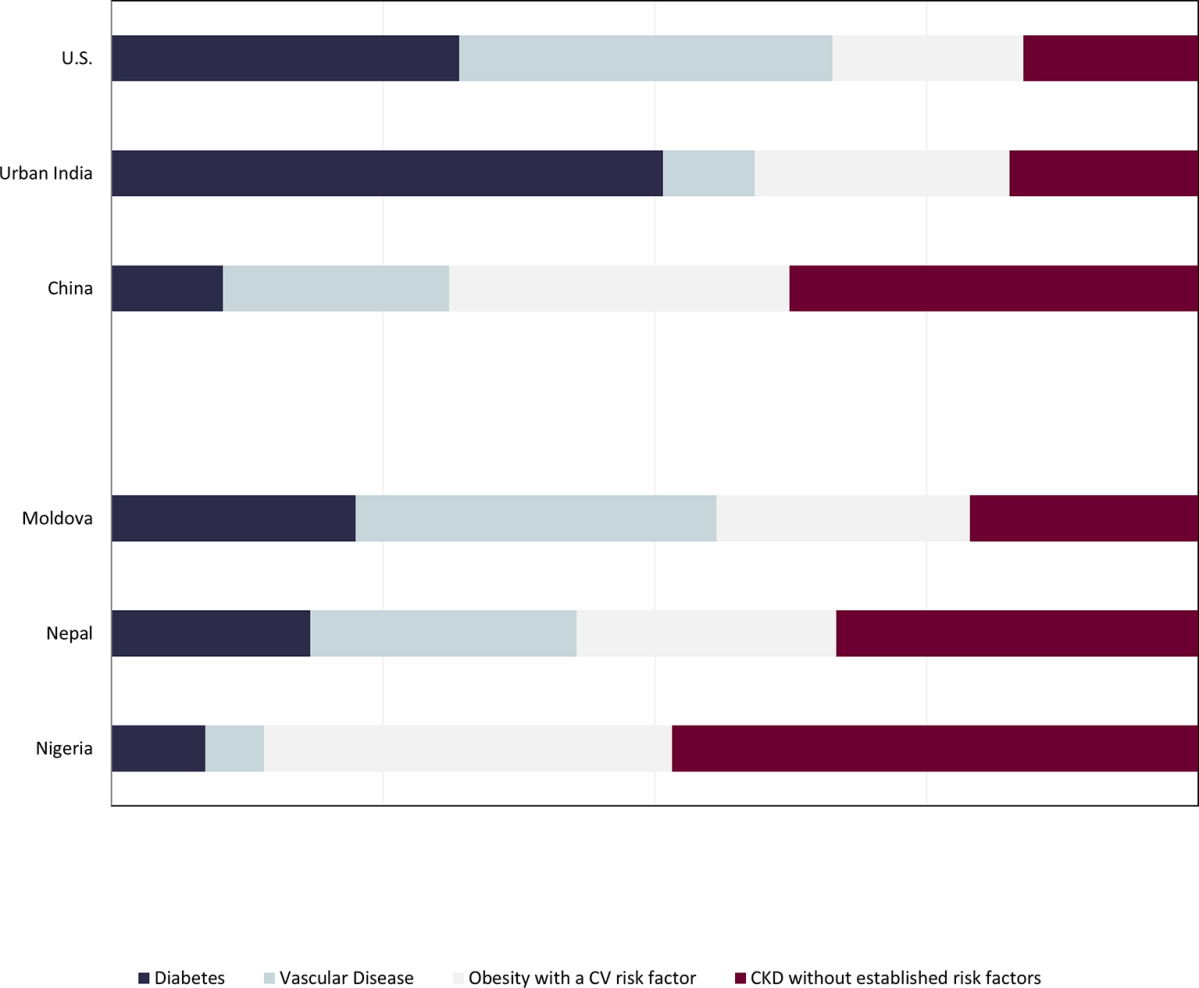
Profiles of persons with CKD. In all countries except Nigeria, a majority of persons (>60%) fit one of the three predefined risk profiles. Diabetes and CKD were most common in urban India; vascular disease and CKD were most common in Moldova; obesity with prehypertension, pre-diabetes or dyslipidaemia was most common in China. CKD, chronic kidney disease; CV, cardiovascular.

Figure 1 depicts the relative prevalence of the four profiles and the overall proportion of persons with CKD in each country fitting these profiles. In the USA, many persons with CKD did fit one of the three profiles with accompanying established risk factors, with only 16.1% (95% CI 13.0% to 19.2%) lacking one of these risk factors. We found similar results in urban India (17.4%, 95% CI 13.4% to 21.4% in profile 4); diabetes mellitus was most common (50.7%, 95% CI 44.9% to 56.6%). Compared with other countries, more Chinese persons with CKD did not have one of the associated established risk factors (37.6%, 95% CI 35.2% to 40.1%), but more than 60% did fit into one of the three profiles, with vascular disease (20.8%, 95% CI 18.7% to 22.9%) more common than in India and obesity with a cardiovascular risk factor most common of the three predefined risk profiles (31.3%, 95% CI 29.0% to 33.6%).

In the KDDC studies, a high proportion of persons with CKD from Moldova could be categorised into one of the three profiles, with vascular disease most common in Moldova (33.2%, 95% CI 27.8% to 38.9%) and prevalence of CKD without established risk factors (21.0%, 95% CI 15.6% to 26.7%) similar to that of USA and urban India. In Nepal, vascular disease (24.5%, 95% CI 22.9% to 26.1%) or obesity with a metabolic risk factor (23.9%, 95% CI 22.3% to 25.5%) was similarly common, and a higher proportion of persons (33.3%, 95% CI 31.7% to 34.7%) had CKD without established risk factors. Nearly half of the participants (48.4%, 95% CI 43.7% to 53.5%) in Nigeria did not have accompanying risk factors.

### Characteristics of persons without established risk factors (profile 4)

**Figure 2:**
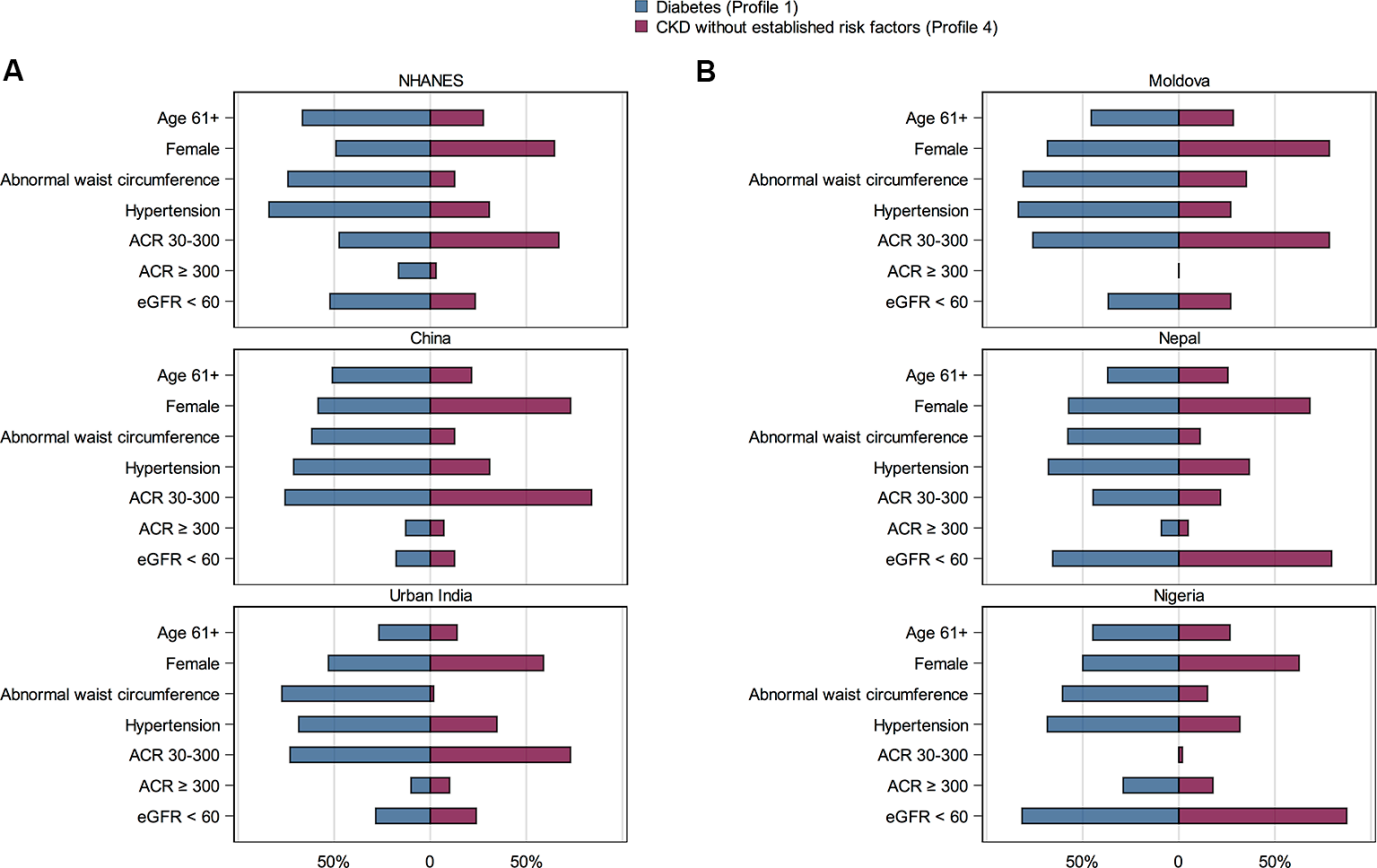
Characteristics of persons with CKD without established risk factors versus those with CKD and diabetes. (A) Among participants of population-based studies. (B) Among participants of International Society of Nephrology Kidney Disease Data Center studies. Persons without established CKD risk factors were younger and more likely female; about one-third had hypertension. Within each country, the distribution of albuminuria and eGFR <60 mL/min/1.73 m^2^ did not differ substantially between the two profiles. ACR, albumin:creatinine ratio; CKD, chronic kidney disease; eGFR, estimated glomerular filtration; NHANES, National Health and Nutrition Examination Survey.

In-depth exploration of harmonised data on persons falling in profile 4 (figure 2A,B) showed that, in contrast to persons with diabetes and associated CKD (profile 1), a majority were less than 61 years in age, with normal waist circumference and without a diagnosis of hypertension. About one-third of persons in profile 4 had hypertension (range of prevalence 27.0% (Moldova) to 35.9% (China)). The distribution of CKD, that is, the proportion of persons with ACRs 30–300 mg/g or with eGFR <60 mL/min/1.73 m^2^, did not seem to vary by profiles within country; significant albuminuria was most common in Nigeria for both profiles 1 and 4.

Supplemental analyses relying solely on fasting glucose thresholds did not demonstrate any significant differences in proportions of persons in profile 4 in NHANES and urban CARRS (online supplementary table 5).

Notably, 10.4% of NHANES participants in profile 4 had missing data for other profiles, a proportion comparable with or higher than other studies (1.1%–11.4%) with the exception of the ISN KDDC study in Moldova. Missingness of waist circumference data was high in this study; thus 27% of persons in profile 4 had missing data for other profiles.

## Discussion

Spanning several regions of the world, our study of persons with CKD demonstrates that a majority have accompanying diabetes mellitus, vascular disease or obesity with a cardiovascular risk factor. While this association does not imply causation, management of the disorders defining these profiles can substantially mitigate risk for end-stage renal disease (ESRD) and cardiovascular events in persons with CKD.^20–22^ Our analysis thus confirms that public health policies in urban India, China, Nepal or Moldova to reduce smoking or improve diet and physical activity or primary care guidelines to optimise glycaemic control are likely to significantly attenuate the downstream burden of CKD. At the same time, the proportion of persons with CKD who do not have established risk factors is larger in LMICs in comparison with the USA, implying that, in order to detect a larger range of CKD, LMICs will need to identify region-specific risk factors (eg, haematuria for regions with high expected rates of IgA nephropathy^23^ or herbal use in regions with medicinal aristolochic acid^24^).

Evidence regarding the prevalence of CKD in LMICs has been growing,^1 2^ but few studies have been performed using a representative sampling technique or with measures of potential attributes. Even in the studies we were able to collate, participants likely under-reported or were underdiagnosed for some profiles. Fewer than 1% of participants in the Nigeria study self-reported cardiovascular disease, for example, and fewer than 7% reported smoking. Yet even if we were to double the proportion of persons with CKD and associated vascular disease (profile 2) in the Nigeria study, nearly 40% would remain in the category of CKD without established risk factors. The high rates of moderate or high albuminuria in this group hint at the influence of apolipoprotein-1 nephropathy,^25^ which may explain why, while the prevalence of diabetes mellitus or vascular disease was relatively low in Nigeria, the prevalence of CKD has been reported to be similar to other LMICs.^26^ The higher proportion of persons with CKD without established risk factors in China and Nepal also deserves further study. In both countries, moderate or high levels of albuminuria were not striking or disproportionate features of ‘profile 4.’ Several non-allopathic Chinese medications used to treat liver, urinary and cardiovascular disease contain aristolochic acid, a known nephrotoxin that causes non-proteinuric CKD.^27^ Infectious aetiologies such as HIV could either directly or indirectly, through complications of treatment, contribute to CKD in persons without other established risk factors.^28^ Unusual CKD cases or causes have been reported elsewhere as well,^29 30^ and a broad brush approach as applied here and suggested by others^8^ has the potential to identify novel risk factors for CKD.

Our approach also highlights the need for studies investigating thresholds for diagnosis of CKD in LMICs. For example, albuminuria is a predominant manifestation of CKD in the two most populous regions in the world (urban India and China), but experts have proposed considering an alternate ACR threshold to define albuminuria in some regions, particularly due to the lower expected creatinine excretion in persons with lower muscle mass or less meat intake—which could falsely increase the ratio. Jafar *et al* 31 showed that the urine albumin-to-creatinine cut-off of ≥30 mg/g had reasonable high correlation with 24-hour urine excretion in the Indo-Asian population. Further studies evaluating persistence of albuminuria and its association with cardiovascular and renal outcomes are crucial to determine how albuminuria relates to cardiovascular and kidney endpoints in LMICs and if region-specific or other varying definitions of albuminuria need to be applied. A similar argument applies to eGFR, for a multitude of reasons but one prominent example: given the lower-protein diets in some regions, perhaps persons would start to experience the higher cardiovascular risk, anaemia or mineral bone disorders at higher levels of serum-creatinine based eGFR than the <60 mL/min/1.73 m^2^ threshold currently applied worldwide.

We do want to emphasise that, in every other country except Nigeria, a substantial majority of persons with CKD seemed to fit one of the three predefined profiles, derived from data on attributes of persons with CKD in HICs. The relative contribution of diabetes and vascular disease differed across the countries and could help to refine the ‘best-buy’ approach to CKD management. As borne out by data from the Million Death Study capturing the increasing number of kidney failure deaths in India and with a lions’ share attributable to diabetes mellitus,^32^ diabetes prevention and treatment would likely substantially reduce the CKD burden in urban India. In Moldova, smoking reduction and vascular risk management may be more relevant. Obesity with a cardiovascular risk factor was consistently associated with between 20% and 30% of persons with CKD across all countries. Performed in a range of ethnicities, several studies have established obesity as a risk factor for incident ESRD.^21 33 34^ We need innovative approaches that integrate CKD detection and triage into the management of obesity, vascular disease and diabetes^35^; limited resources for renal replacement therapy that might be applied to persons who progress to ESRD in LMICs lend greater urgency to this need.

This analysis has several strengths. Most importantly, information was either uniformly collected (for the three LMICs incorporated by the ISN) or easily harmonised. Three of the cohorts were nationally representative (China, urban India and the USA), and those that were not captured have diverse populations in terms of age, sex and other clinical characteristics to allow for reasonable inferences. Sample sizes were reasonably large, allowing for precise estimates of means and proportions. Serum and urine were collected from most participants, which allowed for identification of perso0ns with albuminuria/proteinuria as well as those with reduced kidney function.

There are also several important limitations. In addition to under-reporting of some lifestyle factors (eg, tobacco use) and medical history that were captured only by self-report, the higher prevalence of CKD in the ISN KDDC studies points to a degree of self-selection among participants. Thus, the profile categorisations from these studies may not be fully generalisable but rather may indicate distribution of CKD profiles within country-specific, clinic-based populations. As such, it is possible that proportion of persons with CKD *without* traditional risk factors is higher in the general population than in a population willing to attend screening. Moreover, the ISN KDDC laboratory methods for measurement of creatinine were not standardised, which could introduce bias both for comparison of the CKD prevalence between study sites and for adequate calculation of eGFR with the CKD-EPI, which assumes creatinine standardisation. However, in each of the ISN KDDC countries, creatinine measurement was calibrated according to manufacturing guidelines, which should have prevented major bias. Since urine dipstick, rather than quantitative measures, was the predominant form of proteinuria assessment in Nepal, there is potential for higher misclassification of persons as having CKD^36^ in this study in particular, although proteinuria determined on urine dipstick alone also confers higher risk for progressive kidney disease.^37^

While few data elements were missing, only one serum and one urine sample were obtained. As such, we would expect some misclassification of CKD, which could be mitigated had the programme obtained three or more serum or urine samples. The criteria we used for determining profiles 1, 2 and 3 (diabetes mellitus, vascular disease and obesity with one or more cardiovascular risk factors) are imperfect. Therefore, even if we were to accurately estimate the prevalence of CKD, we could misclassify persons by profile. Differences in missingness could result in misclassification into profile 4 as well; we noted this problem in particular for Moldova, which could mean that potentially even fewer than the currently estimated 21% of persons with CKD would have CKD without established risk factors. Replication of our approach would thus require careful attention to missingness of data elements defining profiles 1–3. Finally, for persons falling in profile 4, we had limited additional information to evaluate potential correlates for CKD (eg, birth weight, occupation, underlying rheumatologic disease, medication use or family history).

In summary, using nationally representative data from three populous countries (one HIC and two LMICs) and uniformly collected data from three smaller LMICs, we categorised persons within three common profiles of CKD. We found that the majority of persons with CKD in each of these widely disparate countries have CKD in association with diabetes mellitus, vascular disease or obesity. Screening and detection efforts could be streamlined by considering these subtypes of risk. Region-specific definitions for CKD markers, novel genetic susceptibilities and/or environmental influences should be considered in regions with higher than expected CKD prevalence and/or where a disproportionate fraction of persons with CKD do not have one of these three risk profiles.
