# Supplementary material for: Do attributes of persons with chronic kidney disease differ in low-income and middle-income countries compared with high-income countries? Evidence from population-based data in six countries
Source: BMJ Glob Health. 2017 Oct 9;2(4):e000453. doi: 10.1136/bmjgh-2017-000453 (PMC5640036; doi:10.1136/bmjgh-2017-000453)
Supplement: Supplementary file 1 [file bmjgh-2017-000453supp001.pdf]

Supplemental Table 1. Available data and laboratory methodology on parameters used to define patient profiles

| Profile Characteristic | U.S. | China | Urban India | Moldova | Nepal | Nigeria |
|---|---|---|---|---|---|---|
| ***Self- report*** | | | | | | |
| Diabetes | X | X | X | X | X | X |
| Coronary artery disease | X | X | X | X | X | X |
| Congestive heart failure | X | | X | | | |
| Stroke | X | X | X | X | X | X |
| Current use of tobacco | X | X | X | X | X | X |
| Former use of tobacco | X | | X | X | X | X |
| Hypertension | X | X | X | X | X | X |
| ***Anthropometry*** | | | | | | |
| Waist circumference | X | X | X | X | X | X |
| Blood pressure | 5 min waiting period, then BP measurement (mercury) x 3 | 5 min waiting period, then BP readings X 3 | 5 min waiting period, then BP measurement x 2. A third BP measurement if the first two > 10 or > 5 mmHg apart for systolic or diastolic respectively. | X | X | X |
| ***Laboratory*** | | | | | | |
| Fasting Glucose | Enzymatic | Enzymatic | Enzymatic | X | X | X |
| A1c | HPLC, Tosoh Automated Glycohemoglobin Analyzer HLC-723G8 | | HPLC (standardized to national glycohemoglobin standardization program) | N/A | N/A | N/A |
| Lipids | Enzymatic | Enzymatic | Enzymatic | N/A (for TG), for HDL: | X | X |
| Urine albumin | Solid-phase fluorescent immunoassay | Immunoturbidimetric | Immunoturbidimetric | | | |

| Serum creatinine | Jaffe rate method (kinetic alkaline picrate), IDMS traceable | Kinetic jaffe method (SCR; calibrated to IDMS after multiplying by 0.95 | Modified (rate blanked, compensated) kinetic jaffe traceable to IDMS | Locally calibrated assay | Locally calibrated assay | Locally calibrated assay |
|---|---|---|---|---|---|---|

Supplemental Table 2. Measures used to categorize albuminuria in participants of the International Society of Nephrology KDDC studies

**MOLDOVA**

| Albuminuria category | < 30 mg/g | | | 30-300 mg/g | | | ≥ 300 mg/g | | | n. rec tot |
|---|---|---|---|---|---|---|---|---|---|---|
| | A/C | P/C | prot dipstick | A/C | P/C | prot dipstick | A/C | P/C | prot dipstick | |
| | 1162 (99.91%) | 0 | 1 (0.09%) | 239 (100%) | 0 | 0 | 1 (100%) | 0 | 0 | |
| | 1163 | | | 239 | | | 1 | | | 1403 |

**NIGERIA**

| Albuminuria category | < 30 mg/g | | | 30-300 mg/g | | | ≥ 300 mg/g | | | n. rec tot |
|---|---|---|---|---|---|---|---|---|---|---|
| | A/C | P/C | prot dipstick | A/C | P/C | prot dipstick | A/C | P/C | prot dipstick | |
| | 0 | 1832 (99.67%) | 6 (0.33%) | 0 | 6 (100%) | 0 | 0 | 68 (100%) | 0 | |
| | 1838 | | | 6 | | | 68 | | | 1912 |

**NEPAL**

| Albuminuria category | < 30 mg/g | | | 30-300 mg/g | | | ≥ 300 mg/g | | | n. rec tot |
|---|---|---|---|---|---|---|---|---|---|---|
| | A/C | P/C | prot dipstick | A/C | P/C | prot dipstick | A/C | P/C | prot dipstick | |
| | 7626 (38.45%) | 5 (0.02%) | 12204 (61.53%) | 551 (54.72%) | 1 (0.10%) | 455 (45.18%) | 4 (1.79%) | 0 | 220 (98.21%) | |
| | 19835 | | | 1007 | | | 224 | | | 21066 |

*A/C= albumin to creatinine ratio; P/C = protein to creatinine ratio; prot dipstick = urine dipstick

Supplemental Table 3. Acceptable ranges for blood pressure, anthropometry, and laboratory values

| Measure | Lower range | Upper range |
|---|---|---|
| Systolic blood pressure (mmHg) | ≥ 60 | ≤ 300 |
| Diastolic blood pressure (mmHg) | ≥ 40 | ≤ 250 |
| Waist circumference (cm) | ≥ 40 | ≤ 200 |
| Fasting Glucose (mmol/L) | ≥ 2.8 | ≤ 28 |
| A1c | ≥ 4 | |
| Triglycerides (mmol/L) | ≥ 0.11 | |
| High density lipoprotein (mmol/L) | ≥ 0.26 | |
| Urine creatinine (micromol/L) | ≥ 0.056 | |
| Serum creatinine (micromol/L) | ≥ 0.003 | |

Ranges are informed by NHANES ranges

**Supplemental Table 4. Characteristics of persons with CKD**

| | U.S. | China | Urban India | Moldova | Nepal | Nigeria |
|---|---|---|---|---|---|---|
| | Mean (se) or % | | | | | |
| Age | 59.04(0.69) | 49.11 (0.46) | 49.26 (1.05) | 56.02 (13.82) | 53.12 (15.47) | 51.31 (11.96) |
| 20-40 | 18.58 | 34.37 | 29.05 | 18.18 | 23.43 | 21.49 |
| 41-60 | 27.08 | 38.71 | 50.04 | 35.23 | 44.25 | 52.04 |
| 61+ | 54.35 | 26.92 | 20.91 | 46.59 | 32.32 | 26.47 |
| Missing | | | | | | |
| Female | 59.35 | 60.93 | 55.08 | 71.88 | 64.08 | 69.68 |
| Missing | | | | | | |
| Current or former smoker | 43.62 | 20.85 | 16.61 | 17.33 | 28.31 | 6.33 |
| Self reported history of cardiovascular disease# | 21.65 | 3.09 | 8.95 | 33.24 | 2.83 | 0.68 |
| Waist circumference (cm) | 103.00(0.74) | 82.14 (0.25) | 89.99 (0.74) | 94.99 (13.62) | 82.32 (11.74) | 85.13 (12.40) |
| Abnormal* | 63.55 | 51.15 | 67.82 | 71.88 | 48.02 | 51.58 |
| Missing | 5.5 | 0.85 | | 7.95 | 0.31 | |
| Systolic blood pressure (mmHg) | 129.98(0.67) | 135.28 (0.61) | 135.44 | 137.83 (22.02) | 129.82 (21.50) | 129.03 (23.73) |
| Missing | 3.5 | 0.13 | 1.63 | 1.7 | | 0.45 |
| <130 | 52.05 | 46.58 | 47.28 | 29.83 | 47.49 | 50.9 |
| 130-<140 | 16.71 | 13.38 | 16.25 | 14.2 | 17.62 | 16.06 |
| ≥ 140 | 27.74 | 39.91 | 34.83 | 54.26 | 34.89 | 32.58 |
| Diastolic blood pressure (mmHg) | 68.67(0.45) | 84.87 (0.33) | 87.60 (0.77) | 86.05 (11.70) | 84.25 (12.97) | 83.00 (15.23) |
| Missing | 5.04 | 0.13 | 1.63 | 1.7 | | 0.45 |
| <85 | 84.51 | 54.77 | 43.77 | 44.6 | 54.19 | 61.09 |
| 85-<90 | 4.99 | 10.02 | 16.45 | 3.41 | 1.9 | 1.13 |
| ≥ 90 | 5.46 | 35.08 | 38.15 | 50.28 | 43.91 | 37.33 |
| Fasting glucose (mmol/L) | 118.02(1.56) | 100.99 (0.84) | 138.75 (3.67) | 99.76 (59.95) | 98.28 (46.19) | 88.53 (35.87) |
| Missing | 0.03 | 0.88 | 0.95 | 3.13 | 0.17 | 8.6 |
| <5.6 | 38.96 | 66.98 | 34.23 | 70.74 | 71.28 | 80.54 |
| 5.6- 6.9 | 39.41 | 23.50 | 31.25 | 9.09 | 14.91 | 7.47 |
| ≥ 7 | 21.59 | 8.64 | 33.56 | 17.05 | 13.64 | 3.39 |
| Hemoglobin A1c | 6.14(0.05) | - | 7.15 (0.11) | - | - | - |

| | | | | | | |
|---|---|---|---|---|---|---|
| <5.7 | 44.24 | - | 2.07 | - | - | - |
| 5.7-6.4 | 33.72 | - | 20.97 | - | - | - |
| ≥ 6.5 | 21.76 | - | 32.74 | - | - | - |
| Missing | 0.28 | - | 44.23 | - | - | - |
| Diabetes^ | 32 | 10.27 | 50.73 | 22.44 | 18.3 | 8.6 |
| Hypertension^ | 67.48 | 48.50 | 56.40 | 69.6 | 54.74 | 46.38 |
| Albumin to creatinine ratio (mmol/g) | | | | | | |
| Missing | 1.99 | 1.94 | 2.25 | | | |
| <3.4 | 36.03 | 6.24 | 15.70 | 33.24 | 71.16 | 83.26 |
| 3.4-<34 | 53.39 | 85.39 | 69.10 | 66.48 | 23.72 | 1.36 |
| ≥ 34 | 8.59 | 6.42 | 12.95 | 0.28 | 5.11 | 15.38 |
| eGFR < 60 ml/min/1.73m$^2$ with or without albuminuria | 44.59 | 12.05 | 27.25 | 43.75 | 80.70 | 89.82 |
| Missing eGFR | 7.74 | 0.21 | - | - | - | - |

Supplemental Table 5. Sensitivity analyses for NHANES and CARRS excluding hemoglobin A1c in profile definitions

| | NHANES | 95% CI | | Urban India | 95% CI | |
|---|---|---|---|---|---|---|
| Including A1c in Profile 1 and 3 definition | | | | | | |
| Profile 1 | 32.0 | 28.8 | 35.2 | 50.7 | 44.9 | 56.6 |
| Profile 2 | 34.3 | 30.9 | 37.7 | 8.5 | 5.4 | 11.5 |
| Profile 3 | 17.6 | 14.5 | 20.6 | 23.4 | 19.2 | 27.7 |
| Profile 4 | 16.1 | 13.0 | 19.2 | 17.4 | 13.4 | 21.4 |
| Excluding A1c in Profile 1 and 3 definitions | | | | | | |
| Profile 1 | 30.6 | 27.3 | 33.9 | 40.5 | 35.3 | 45.6 |
| Profile 2 | 35.2 | 31.9 | 38.5 | 10.9 | 7.7 | 14.1 |
| Profile 3 | 17.8 | 14.5 | 21.0 | 29.4 | 25.0 | 33.7 |
| Profile 4 | 16.4 | 13.3 | 19.6 | 19.3 | 15.1 | 23.5 |
